# Seed Coating: A Tool for Delivering Beneficial Microbes to Agricultural Crops

**DOI:** 10.3389/fpls.2019.01357

**Published:** 2019-11-06

**Authors:** Inês Rocha, Ying Ma, Pablo Souza-Alonso, Miroslav Vosátka, Helena Freitas, Rui S. Oliveira

**Affiliations:** ^1^Centre for Functional Ecology – Science for People & the Planet, Department of Life Sciences, University of Coimbra, Coimbra, Portugal; ^2^Department of Mycorrhizal Symbioses, Institute of Botany, Academy of Sciences of the Czech Republic, Pru˚honice, Czechia

**Keywords:** arbuscular mycorrhizal fungi, plant growth-promoting bacteria, rhizobia, seed coating, sustainable agriculture, *Trichoderma*

## Abstract

Plant beneficial microbes (PBMs), such as plant growth-promoting bacteria, rhizobia, arbuscular mycorrhizal fungi, and *Trichoderma*, can reduce the use of agrochemicals and increase plant yield, nutrition, and tolerance to biotic–abiotic stresses. Yet, large-scale applications of PBM have been hampered by the high amounts of inoculum per plant or per cultivation area needed for successful colonization and consequently the economic feasibility. Seed coating, a process that consists in covering seeds with low amounts of exogenous materials, is gaining attention as an efficient delivery system for PBM. Microbial seed coating comprises the use of a binder, in some cases a filler, mixed with inocula, and can be done using simple mixing equipment (e.g., cement mixer) or more specialized/sophisticated apparatus (e.g., fluidized bed). Binders/fillers can be used to extend microbial survival. The most reported types of seed coating are seed dressing, film coating, and pelleting. Tested in more than 50 plant species with seeds of different dimensions, forms, textures, and germination types (e.g., cereals, vegetables, fruits, pulses, and other legumes), seed coating has been studied using various species of plant growth-promoting bacteria, rhizobia, *Trichoderma*, and to a lesser extent mycorrhizal fungi. Most of the studies regarding PBM applied *via* seed coating are aimed at promoting crop growth, yield, and crop protection against pathogens. Studies have shown that coating seeds with PBM can assist crops in improving seedling establishment and germination or achieving high yields and food quality, under reduced chemical fertilization. The right combination of biological control agents applied *via* seed coating can be a powerful tool against a wide number of diseases and pathogens. Less frequently, studies report seed coating being used for adaptation and protection of crops under abiotic stresses. Notwithstanding the promising results, there are still challenges mainly related with the scaling up from the laboratory to the field and proper formulation, including efficient microbial combinations and coating materials that can result in extended shelf-life of both seeds and coated PBM. These limitations need to be addressed and overcome in order to allow a wider use of seed coating as a cost-effective delivery method for PBM in sustainable agricultural systems.

## Introduction

Currently, more than 1/3 of the Earth’s land surface is occupied by agriculture, with a total net production value estimated in 2.6 × 10^9^ US dollar (FAOSTAT, 2016). Only in Europe, there are 20 million people working regularly in the agricultural sector, with 44 million jobs currently taken in farming and the food sector (European Commission, 2019). It is a sector of great importance, providing employment for about 50% of the labor force in low-income countries (Cheong et al., 2013) and being the basis of human dietary composition (Awika, 2011). Nonetheless, the unsustainability of the current conventional agricultural practices along with future climate scenario urges for alternatives that can not only increase agricultural production but also bring environmental and economic sustainability, thus ultimately improving human well-being (Gliessman, 2005; Reganold and Watcher, 2016).

Plant beneficial microbes (PBMs) are considered to be a natural alternative path to ease the pressure on the environment resulting from conventional farming. These microbes can help plants maintain or increase productivity while reducing the input of agrochemicals, restoring soil fertility, and/or overcoming problems caused by abiotic and biotic stresses (Malusá et al., 2012; Nadeem et al., 2014). In the last decades, the interest in the use of PBM for increasing yields and resilience of agricultural crops has been growing steadily (Jeffries et al., 2003; Royal Society of London, 2009; Hayat et al., 2010; Li et al., 2014; Vurukonda et al., 2016). Yet, agricultural practices such as intensive fertilization and soil tillage and abusive application of pesticides can severely affect soil microbes and their beneficial interaction with the target plants, thus hampering a wider use of PBM (Lupwayi et al., 2012; Tsiafouli et al., 2015; Prashar and Shah, 2016). Moreover, currently, the large-scale application of PBM, particularly in broad-acre crops, might not be practicable or economically feasible considering the amount of microbial inoculum needed per plant (Vosátka et al., 2012; Oliveira et al., 2016a). Therefore, there is an urgent necessity of efficient and effective inoculation methods to apply PBM (Glick, 2012)

Seed inoculation has been considered as a precise and cost-effective method to deliver microbial inoculants (Ehsanfar and Modarres-Sanavy, 2005; O’Callaghan, 2016), with the potential for large-scale application. Seed coating is a technique in which an active ingredient (e.g., microbial inoculant) is applied to the surface of the seed with the help of a binder and in some cases a filler that can act as a carrier. Seed coating has been proposed as a promising tool for inoculation of different crop seeds, since it is able to use minor amounts of inocula in a precise application (Jetiyanon et al., 2008; Oliveira et al., 2016a; Rouphael et al., 2017; Accinelli et al., 2018a; Accinelli et al., 2018b; Rocha et al., 2019a; Rocha et al., 2019b). The main types of seed coatings include seed dressing, film coating, and pelleting, which can be chosen differently, according to the purpose of application and the type of seed or selected microbes.

In this review, we considered seed coating as any method in which the seed surface is covered by materials (solid or liquid containing dissolved or suspended solids) forming a more or less continuous layer (physical barrier). Applications without the purpose of seed covering, which only comprise the use of microbial inoculants lacking any other compounds such as fillers/carriers or binders, like bacterization by seed immersion in a bacterial suspension (seed soaking) were considered as seed treatments but not seed coating.

Here, published research on microbial inoculants applied *via* seed coating is reviewed, with the intention of evaluating the effectiveness of seed coating as a delivery system for microbial formulations and their effects on agricultural crops. New research opportunities and future prospects are also highlighted.

## Plant Beneficial Microbes

Microorganisms that benefit plant establishment, growth, and development by direct or indirect mechanisms are generally known as PBM. This review mostly focuses on two main groups of soil microorganisms, bacteria and fungi, particularly on plant growth-promoting bacteria (PGPB), arbuscular mycorrhizal (AM) fungi, and *Trichoderma*, due to their importance as microbial inoculants in agroecosystems.

### Bacteria

Bacteria are, by far, the most abundant microorganisms present in the rhizosphere (Kaymak, 2010). Various genera of bacteria (e.g., *Azospirillum*, *Azotobacter*, *Pseudomonas*, *Bacillus* and *Burkholderia*) contain species that have positive effects on plant growth and development. These beneficial bacteria, also designated as PGPB, are responsible for protecting plants from biotic and abiotic stresses, enhancing plant growth and performance through direct and indirect mechanisms (Glick, 1995; Timmusk et al., 2017). PGPB can act as biofertilizers, phytostimulators, rhizoremediators, stress bioalleviators, biomodifiers, or biological control agents (BCAs)/biopesticides (Lugtenberg and Kamilova, 2009; Ma et al., 2016). Direct mechanisms include facilitation of nutrient acquisition [e.g., nitrogen (N) fixation, phosphorus (P) and potassium (K) solubilization], synthesis of phytohormones (e.g., indole-3-acetic acid and cytokinins), and production of ammonia, organic acids, and 1-aminocyclopropane-1-carboxylate (ACC) deaminase (Hayat et al., 2010; Glick, 2012; Bulgarelli et al., 2013; de Souza et al., 2015; Vejan et al., 2016). On the other hand, indirect mechanisms limit or prevent plant damage and they include biological control against phytopathogens (e.g., bacteria, fungi, nematodes), mainly through the synthesis of allelo chemicals (e.g., antibiotics, hydrogen cyanide) and lytic enzymes, as well as the activation of induced systemic resistance (Compant et al., 2005; Bakker et al., 2007; Glick, 2012). Despite not traditionally considered as PGPB, rhizobia is the term commonly used to describe bacteria capable of nodulation and N_2_ fixation in association with leguminous plants (Willems, 2006). Moreover, rhizobia are also capable of colonizing the roots of non-legumes and producing phytohormones, siderophores, and hydrogen cyanide and showing antagonistic effects against certain plant pathogenic fungi (Glick, 2012). Inoculation of rhizobia onto legume seeds is probably the oldest agrobiotechnological application (Lindström et al., 2010). Responsible for an essential N contribution to agroecosystems, the application of rhizobia is a common agricultural practice and several commercial inoculants containing PBM are based on rhizobia formulations (Xavier et al., 2004; Benrebah et al., 2007). Once established, inoculated plants significantly reduce synthetic N requirements (30%–60%) in comparison with conventional crops (Jensen et al., 2012).

### Fungi

Other important PBMs are AM fungi and *Trichoderma*. AM fungi associate with the roots of almost 80% of terrestrial plants to form arbuscular mycorrhizas (Harley and Smith, 1983). These symbiotic associations are of great relevance for agricultural systems especially under low input of agrochemicals, due to their role in increasing nutrient uptake and acquisition (e.g., up to 80% of the acquired P can be supplied by the mycorrhiza) (Jeffries et al., 2003; Gąstoł et al., 2016; Oliveira et al., 2016b). Moreover, AM fungi are able to improve soil aggregation, provide a protective barrier against pathogens, and increase water acquisition (Rillig and Mummey, 2006; Sikes, 2010; Nadeem et al., 2014; Bücking and Kafle, 2015; Oliveira et al., 2016b). Besides the structural and nutritional benefits, AM fungi can help crops cope with environmental stresses, therefore enhancing plant growth by producing metabolites (e.g., amino acids, vitamins, phytohormones, and antioxidant enzymes) and adjusting plant physiological status (e.g., proline content, carbon dioxide exchange rate, and stomatal conductance) (Evelin et al., 2009; Ruiz-Lozano and Aroca, 2010; Sikes, 2010; Birhane et al., 2012; Nadeem et al., 2014). For instance, different AM fungal species [e.g., *Glomus intraradices*, *Rhizophagus irregularis*, *Glomus mosseae* (renamed to *Funneliformis mosseae*), and *Rhizophagus fasciculatus*] have been used to improve crop performance under salinity and drought stresses (Abdul Latef and Chaoxing, 2011; Celebi et al., 2010; Alizadeh et al., 2011; Habibzadeh et al., 2013; Li et al., 2014; Oliveira et al., 2017a; Oliveira et al., 2017b; Chun and Chandrasekaran, 2018).

As common free-living fungi in the rhizosphere and soil, *Trichoderma* spp. are well known for their ability to produce a wide range of antibiotics and to parasitize other fungi. Metabolites released during plant–*Trichoderma* interaction (see Contreras-Cornejo et al., 2016 for a detailed list of active metabolites) can influence several aspects of plant development such as plant growth and root morphology and nutritional status (increase in nutrient uptake, N use efficiency, and nutrient solubilization), and trigger induced systemic resistance, biocontrol of pathogens, and inactivation of toxic compounds in the root zone. Characteristics like high resilience, aggressiveness, reproductive capacity, and efficient nutrient exploitation contribute for *Trichoderma* successful establishment in the rhizosphere (Harman et al., 2004). Other fungi (e.g., *Aspergillus* spp., *Beauvaria bassiana*, and *Gliocladium virens*) and the oomycete *Pythium oligandrum* used as biofertilizers and BCA applied *via* seed coating are also mentioned in this review.

### Microbial Consortia

Interactions between different PBM and host plants can be fundamental to maintain soil fertility and plant health, particularly in low-input agriculture that relies on biological process rather than agrochemicals (Sessitsch and Mitter, 2015). Combinations of different PBM, as microbial consortia, can result in improved plant performance. PGPB have been shown to positively influence legume–rhizobia and plan–fungi interactions (Vessey, 2003; Mohamed et al., 2014; Korir et al., 2017). The combined use of PGPB and N-fixing bacteria can improve root growth and plant resilience to environmental stresses, and reduce N losses (Dal Cortivo et al., 2017). It is well known that PGPB can be used to ameliorate nodule formation in legumes when co-inoculated with rhizobia (Tilak et al., 2006) and enhance plant growth indirectly by optimizing the relationship between host plants and AM fungi. Ratti et al. (2001) found that *Bacillus polymyxa* and *Azospirillum brasilense* enhanced root colonization by *Glomus aggregatum* and improved biomass and P content of palmarosa grass when grown on insoluble inorganic phosphate source. Moreover, AM fungi can also associate with legumes where rhizobia are present to increase grain yield and protein content (Oliveira et al., 2017a; Oliveira et al., 2017b). For example, a consortium of *G. mosseae* and *Trichoderma harzianum* increased the yield and seed quality of different agricultural crops (Egberongbe et al., 2010; Nzanza et al., 2012). Notwithstanding, the application of microbial consortia does not necessarily entails positive interactions. Competition for nutrient and niche and production of antagonistic secondary metabolites can occur. Therefore, the selection of appropriate PBM to be applied in consortia is crucial.

## Microbial Inoculation

PBMs are usually added to the soil (direct soil application), the seed (seed-applied inoculant), or the plant (e.g., foliar spray and root dipping) (Adholeya et al., 2005; Mahmood et al., 2016). Each inoculation method has advantages and disadvantages, depending on the amount of inoculants, availability of equipment, type of seed (e.g., size, shape, and fragility), the presence of inhibiting compounds in the seed (e.g., fungicides, micronutrients, and PBM), and cost (Deaker et al., 2004; Bashan et al., 2014). A summary of some of the most common techniques used in the different inoculation methods and their advantages and disadvantages is presented in **Table 1**.

**Table 1:** Methods of application of microbial inoculants.

Method	Technique	Advantages	Disadvantages	References
**Direct soil inoculation**	Granular/powder; liquid inoculation; immobilized microbial cells	Avoids damaging fragile seeds and cotyledons; overcomes the adverse effect of pesticides and fungicides applied to seed; small seeds can receive higher dose of inoculant	Requires specialized equipment for application and larger quantities of inoculants; requires more storage area and transport; expensive method	Van Elsas and Heijnen, 1990; Smith, 1992; Deaker et al., 2004; Adholeya et al., 2005; Bashan et al., 2014
**Plant inoculation**	Foliar spray; root dipping	Direct application; application of microbial inoculant with high concentration	Expensive; requires large amount of inoculant; laborious and time consuming	Adholeya et al., 2005; Mahmood et al., 2016
**Seed inoculation**	Seed soaking; seed coating (seed dressing; film coating; pelleting/encrusting; slurry coating); bio-priming	Practical and ready-to-use product; fast, cheap and accurate; require low amount of inoculant; confers other beneficial characteristics to the seed	Poor survival of the inoculant (reduced shelf-life); insufficient amount of microbial inoculant for small seeds (except for pelleting); incompatibility of seeds treatments (e.g., fungicides); seed coat lifted out of the soil during germination	Kaufman, 1991; Smith, 1992; Adholeya et al., 2005; Ehsanfar and Modarres-Sanavy, 2005; Deaker et al., 2012; Bashan et al., 2014; Mahmood et al., 2016

In general, direct soil inoculation is used to introduce a large amount of microbial inoculant into the soil, avoiding damage of fragile seeds or protecting the inoculant from inhibiting compounds applied or produced by the seed (e.g., fungicides and antimicrobial compounds). It can be done either using solid, liquid, or encapsulated formulations at the time of seeding (Malusá et al., 2012; Bashan et al., 2014). However, direct soil inoculation is not economically feasible in large-scale applications due to the high amount of microbial inoculum required (Deaker et al., 2004; Adholeya et al., 2005; Vosátka et al., 2012).

Although inoculation of plants through root dipping and foliar is currently being used, these techniques demand large amounts of inoculant and, in the case of root dipping, plant nursery preparation is also required. On the other hand, seed inoculation can be a cost-effective way to deliver microbes in large-scale field applications (John et al., 2010; O’Callaghan, 2016). Seed inoculation delivers PBM to the rhizosphere of the target crop, where an intimate plant–microbe contact is established since germination (Philippot et al., 2013). Besides being a precise delivery system, seed inoculation can also be used to modify seed characteristics (e.g., shape, size and weight, etc.), making it easy to handle and sow (Halmer, 2008).

PBM inoculation has potential to be applied worldwide, from small- to large-scale agricultural systems. Considering that about 90% of the world’s farms are held and ran by families, most of them small and found in rural areas of developing countries (FAO, 2018), it is essential to apply PBM using affordable techniques with simple technological setup. For instance, N2Africa was a project created to promote the use of rhizobia through seed inoculation among smallholder farmers growing legume crops (e.g., common bean, chickpea, cowpea, and soybean) in 11 countries of sub-Saharan Africa (e.g., Ethiopia, Kenya, Mozambique, Nigeria, Rwanda) (Woomer et al., 2017). More than promoting the use of rhizobia, the project intended for knowledge dissemination, focusing in linking scientific research with capacity building, training researchers, students, farmers, and decision-makers to develop and implement sustainable strategies for agriculture.

## Seed Coating With Beneficial Microbes

Seed coating is the application of exogenous materials onto the surface of seeds with the aim of improving seed appearance and handling characteristics (e.g., seed weight and size) and/or delivering active compounds (e.g., plant growth regulators, micronutrients, and microbial inoculants) that can protect the seed against phytopathogens and increase germination and plant growth (Halmer, 2008; Pedrini et al., 2017). Inspired in the pharmaceutical industry, seed coating was first applied to cereal seeds in the 1930s, and thereafter, its large-scale commercial use began in the 1960s (Kaufman, 1991). Nowadays, seed coating is used by horticultural and crop industries worldwide and has earned its place in the global market (Pedrini et al., 2017). It is used for applying colors and tracers (e.g., fluorescent dyes); protectants (e.g., pesticides); soil adjuvants (e.g., soil hydrophilic materials and hydro-absorbers); compounds that stimulate germination, growth, and stress resistance (e.g., salicylic acid, gibberellic acid, and abscisic acid); and macronutrients and micronutrients and PBM inoculants (Scott, 1989; Ehsanfar and Modarres-Sanavy, 2005; Pedrini et al., 2017). Coating crop seeds with PBM allows a precise application of minor amounts of inocula at the seed–soil interface (Scott, 1989), ensuring that the PBMs are readily accessible at germination and early development plant stages, stimulating healthy and rapid establishment, and consequently maximizing crop production (Colla et al., 2015a).

### Ingredients, Types, and Equipment

Seed coating can vary from simple on-farm applications to sophisticated and industrialized procedures. Although the processes used by farmers and industrial companies may differ, the principle is basically the same. Overall, it includes, seeds inside a container (e.g., rotating drum, cement mixer), where a binder (e.g., adhesive compound), a filler (bulking agent) if needed, and active ingredients (e.g., nutrients, protectants, and PBM) are mixed (Scott, 1989; Accinelli et al., 2016; Padhi and Pattanayak, 2018). Fillers can be single or mixed components, and the most commonly applied are peat (Georgakopoulos et al., 2002; Hartley et al., 2004; Hameeda et al., 2010), talc (Mukherjee and Sen, 1998; Sabaratnam and Traquair, 2002; Berninger et al., 2016), and lime (Brockwell and Phillips, 1970; Gault and Brockwell, 1980; Padhi and Pattanayak, 2018). These components can function as microbial carriers and modify seed size, shape, and weight. Some ingredients like alginate can be used both as filler and binder (Heo et al., 2008; Khan et al., 2011; Anis et al., 2012; Lally et al., 2017). Recently, biochar and chitosan have been also considered as fillers/carriers for microbial seed coating (Głodowska et al., 2016; Głodowska et al., 2017; Ruiz-de-la-Cruz et al., 2017). Binders, natural or synthetic polymers such as methyl cellulose (Hartley et al., 2004; Haikal, 2008; Swaminathan et al., 2016; Amutha, 2017; Lopisso et al., 2017), carboxymethyl cellulose (Sharma et al., 2003; Roesti et al., 2006; Nawar, 2007; Zhou et al., 2017), gum arabic (Kyei-Boahen et al., 2001; Ehteshamul-Haque et al., 2007; Dawar et al., 2008; Singh et al., 2014), or polysaccharide Pelgel (Jensen et al., 2000; Li et al., 2002; Ugoji et al., 2006) are generally added during or toward the end of the coating process in order to bind the exogenous materials and reduce the amount of dust in the final product (Pedrini et al., 2017). Some adhesives (e.g., gum arabic and xanthan gum) can also be used to extend the survival of PBM applied to seeds (Jambhulkar et al., 2016). The selection of the proper type and concentration of binder and filler is crucial for seed germination, plant development, and viability of the applied microbial inoculant. Other characteristics such as availability, cost, origin, and environmental impacts should also be taken into consideration when choosing the most adequate coating materials.

The classification of seed coating types is usually based on the weight, size, and grouping properties of the coated seeds. Most studies do not specify the type of coating used, yet when reported, the most frequent are seed dressing, film coating, and pelleting (Hartley et al., 2004; Shaharoona et al., 2008; Domaradzki et al., 2012; Accinelli et al., 2016; Celly et al., 2016; Jacob et al., 2016; Shahzad et al., 2017; Accinelli et al., 2018b; Rocha et al., 2019a). Moreover, other terms such as slurry coating can also be found in the literature related to microbial seed coating (Pill et al., 2009; Hartley et al., 2013; Rozier et al., 2017; Rehman et al., 2018).

The most basic coating treatment is seed dressing, which refers to the application of finely milled solids dusted onto the surface of seeds in small amounts, and it is normally used for pesticide application (Scott, 1989). Yet, some studies use the term seed dressing, not as a type but as synonym for seed coating (Shaharoona et al., 2008; Choi et al., 2016; Murphy et al., 2017; Shahzad et al., 2017). Film coating is considered as a more recent method, and it consists on the application of a thin layer of external material with little change of the seed shape, size, and weight (Halmer, 2000; Halmer, 2008). It can be considered an improved version of slurry coating, where a solution or suspension is also applied onto the seeds, but in a less firm and uniform layer (Taylor et al., 2001; O’Callaghan, 2016). Also, film coating allows better treatment precision and minimizes the production of dust. It is considered a well-established technique for coating of several high-value horticultural species and other important agricultural crops, such as maize, sunflower, soybean, and canola (Accinelli et al., 2016). In comparison with other seed coating types, film coating has a lower interference with seed germination and a prompter release of active components (Halmer, 2008). Finally, pelleting comprises fillers and liquid binders applied to the seed that may cause a significant increase in weight and volume. Pelleting usually modifies seed morphology into a spherical or ovoid shape, making it impossible to discriminate the initial seed shape (Halmer, 2000). If the original seed shape is still maintained, the term used is encrusting (Pedrini et al., 2017). Pelleting and encrusting increase the amount of applied active ingredients and improve seed handling and sowing, especially for irregularly shaped seeds (Halmer, 2008).

Depending on the type of coating, specific equipment is considered [for some examples, see Pedrini et al. (2017)]. The rotating pan is the most common device used for seed coating (e.g., pelleting, encrusting, dressing, and film coating) (Hartley et al., 2013; Oliveira et al., 2016a; Rouphael et al., 2017; Accinelli et al., 2018a; Rocha et al., 2019a; Rocha et al., 2019b). It usually consists of an inclined round pan rotating in slow motion, where materials are gradually added, followed by size sorting (sieving and screening) and then drying (Halmer, 2000; Pedrini et al., 2017). Film coating and encrusting can also be carried out using a fluidized or spouted bed, a cylindrical apparatus where seeds are kept in suspension by a constant vertical/bottom-up hot airflow, while being sprayed with coating materials. The warm airflow allows moisture evaporation. This is a slow and costly process (Robani, 1994). Another device used for most seed coating types is the rotary coater or rotor–stator, a cylindrical drum with two rotating base disks, a concave one, whose rotation causes seeds to move steadily along the drum walls, and a smaller one that allows the atomization and projection of liquid/slurry coating to the rotating seed mass (Pedrini et al., 2017). Unfortunately, the majority of scientific publications disclose scarce information regarding equipment and methodological details, with a considerable number reporting seed coating procedures performed by specialized companies (Ugoji et al., 2006; Diniz et al., 2009; Junges et al., 2013; Rozier et al., 2017).

### Formulation and Microbial Survival

The formulation of microbial inoculants generally consists of three basic elements: the selected microorganism, a suitable carrier (that can be solid or liquid), and different additives. It is worth to note that factors such as incorrect inoculant formulation or limited shelf-life (i.e., inoculant viability on the seed surface) can hamper a wider use of seed coating (O’Callaghan, 2016). Formulation has a major impact on the microbial survival during the process of product elaboration, storage, and application, in its efficiency once applied on the target plant and in the economic feasibility of the application (John et al., 2011; Herrmann and Lesueur, 2013). Although the formulation of microbial inoculants is a critical issue, little research on this topic has been conducted (Parnell et al., 2016). Georgakopoulos et al. (2002) evaluated pre-selected bacterial and fungal antagonists responsible for biological control of damping-off in sugar beet and cucumber with the intention of developing potential commercial formulations based on a peat carrier material for seed coating. *Pseudomonas* antagonists were the most effective biocontrol agents and survived for 2 years at ambient temperature in the peat formulation. Moreover, a biochar-based seed coating with *Bradyrhizobium japonicum* inoculum allowed the maintenance of a high bacterial population for over 4 months, which ensured efficient nodulation of soybean (Głodowska et al., 2017). Bacterial survival was strongly affected by the physical and chemical properties of biochar. In fact, out of five applied biochar carriers, only two provided suitable conditions to maintain bacterial viability for long periods of time (9 months). On the other hand, alginate beads can also be used as carriers, which allow a slow and constant release of bacteria. Bashan (1986) developed synthetic beads made of sodium alginate and skim milk, which are biodegradable and have no negative impact on the environment. The final product that consists of lyophilized beads containing immobilized bacterial inoculants can be coated onto crop seeds and then stored at ambient temperature at least for 3 months without loss of bacterial viability. Under high-humidity conditions and without any drying procedure, coated seeds with the immobilized bacteria maintained high viability; however, the downside was that seeds germinated before sowing. Maintaining the viability of PBM coated onto seeds can be challenging, but it is essential for commercial applications. Nevertheless, the shelf-life of seeds coated with microbial inoculants, including the viability of both seeds and coated microbes, is still an overlooked topic in the literature.

### Delivery of Beneficial Microbes

**Figure 1:**
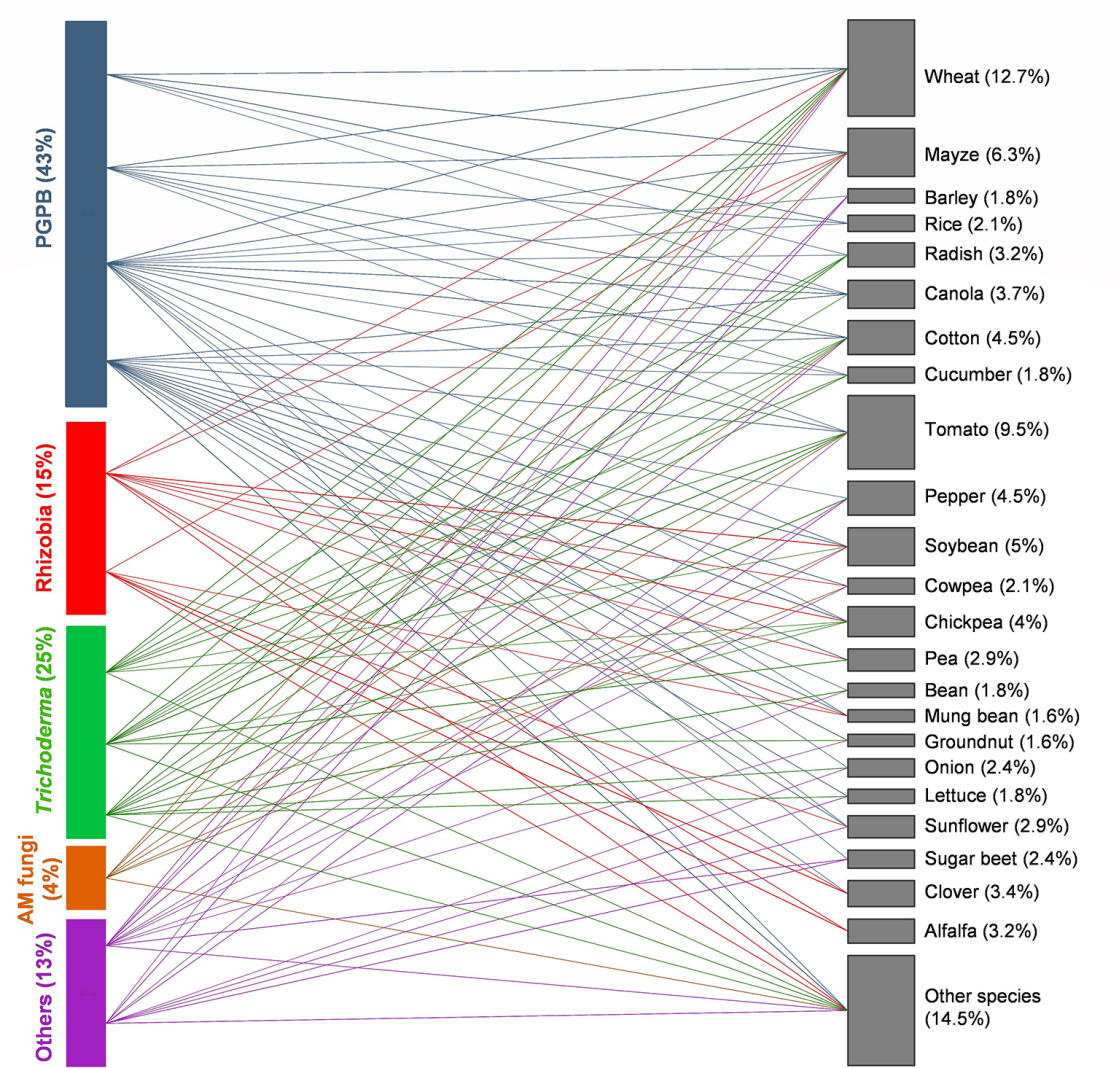
Bipartite network of interactions between plant beneficial microbes (PBMs) and agricultural crops (from a total of 191 papers published between 1960 and 2019). Each colored line represents a specific association. In each case, the size of boxes is proportional to the number of interactions considered (a single study can include several interactions). Plant growth-promoting bacteria (PGPB) (blue), Trichoderma (green), rhizobia (red), arbuscular mycorrhizal (AM) fungi (yellow), and others [fungi (e.g., Aspergillus spp., Beauvaria bassiana) and the oomycete Pythium oligandrum] (purple). Percentages represent the proportion of interactions where the specific groups of PBM or plant species are participating.

An analysis of the published literature since 1960 has showed that a great majority of studies on microbial seed coating were conducted with PGPB (**Figure 1**). Rhizobia and *Trichoderma* are also among the most studied microbial inoculants. Within PGPB, *Pseudomonas* and *Bacillus* are the most commonly applied genera, which are mainly used as plant growth promotors (Bashan, 1986; Junges et al., 2013; Kumar et al., 2015; Choi et al., 2016; Głodowska et al., 2016; Rehman et al., 2018) and BCA (Georgakopoulos et al., 2002; Pereira et al., 2007; Sim et al., 2008; Singh et al., 2012; Moussa et al., 2013; Zhou et al., 2018). Co-coating of *Pseudomonas* and *Bacillus* increased seed vigor and decreased the infection level of *Xanthomonas oryzae* pv. *oryzae* in rice (Palupi et al., 2017) and enhanced canola height and biomass under greenhouse and field conditions (Lally et al., 2017). As the most frequently used rhizobial genus, *Rhizobium* has also been successfully coated singly and in consortia with other PBM, which resulted in positive effects on plant growth and yield (Fatima et al., 2006; Dawar et al., 2008; Dal Cortivo et al., 2017; Zhou et al., 2017; Padhi and Pattanayak, 2018). In some cases, the application of a certain ingredient for seed coating can limit the positive role of *Rhizobium* in plants. Adams and Lowther (1970) assessed the combined effect of lime and *Rhizobium* spp. *via* direct soil inoculation and seed coating on the establishment and growth of different clover species. Direct soil inoculation significantly increased nodulation and caused a threefold rise in plant yield after 32 weeks. Lime also greatly improved nodulation and yield with less intensity compared to direct soil inoculation. Yet, coating of inoculated seeds with lime had little or no effect on clover nodulation or yield. In fact, inoculated seeds coated with lime seemed to display reduced rhizobial survival. Similarly, the application of certain fungicides [e.g., N-(tri-chloromethylthio)-4-cyclohexene-1,2-dicarboximide, metalaxyl-M, carbathiin, oxycarboxin, and thiram] to seeds can be harmful to *Rhizobium* spp. depending on the species or strain, bacteria–fungicide contact period prior to planting, fungicide concentration, and environmental variables (e.g., high temperatures and dehydration). The survival of *Rhizobium ciceri* that was coated onto chickpea seeds and simultaneously treated separately with four commercial fungicides under laboratory conditions was reduced, according with the applied fungicide. In pot experiments, the negative effects of fungicides on *Rhizobium* sp. were less intense, due to the buffer effect of the rhizosphere soil or the possible migration of inoculated strains from the fungicide zones. Kyei-Boahen et al. (2001) described discrepancies between the obtained results and previous reports and highlighted the importance of selecting an adequate fungicide compatible with the specific *Rhizobium* strain for seed coating application. Despite its ability to increase plant productivity and nutrition under greenhouse experiments (Oliveira et al., 2016a; Rocha et al., 2019a) and yield of different agricultural crops under field conditions (Cely et al., 2016), the potential of AM fungi inoculation *via* seed coating to enhance plant performance is still poorly explored (**Figure 1**). On the other hand, as the most used group of fungi for seed coating, *Trichoderma* shows great ability to increase seed germination and plant growth (Nawar, 2007; Domaradzki et al., 2012; Accinelli et al., 2016), and control pathogenic agents such as *Rhizoctonia solani* (Mihuta-Grimm and Rowe, 1986; Dawar et al., 2008; Haikal, 2008), *Pythium* spp. (Hadar et al., 1984; Sivan et al., 1984; Lifshitz et al., 1986; Taylor et al., 1991), *Sclerotium cepivorum* (McLean et al., 2005), and *Fusarium* spp. (Sivan and Chet, 1986; Sivan et al., 1987; Babychan and Simon, 2017) under greenhouse and field conditions. For instance, simultaneous seed coating with inocula of *G. intraradices*, *G. mosseae*, and *Trichoderma atroviride* enhanced growth, nutrient uptake, grain yield, and quality of winter wheat (Colla et al., 2015a). Other fungi such as *Aspergillus* spp. and *G. virens* were inoculated *via* seed coating mainly for biocontrol purpose (Dawar et al., 2008; Haikal, 2008; Singh et al., 2012). Combining different PBM in consortia can improve plant growth and performance (Nadeem et al., 2014). However, only 19% of studies (from a total of 191 papers published between 1960 and 2019) used seed coating with more than one type of PBM. Singh et al. (2014) developed chickpea seed coating with different combinations of *Pseudomonas aeruginosa* PHU094, *T. harzianum* THU0816, and *Mesorhizobium* sp. RL091 using gum arabic as a binder. The aim was to evaluate the effectiveness and potential of the PBM to promote plant growth and phenolic acid biosynthesis in chickpea infected with the fungal pathogen *Sclerotium rolfsii*. The consortium led to superior plant growth and higher amounts of phenolic compounds in chickpea grown under biotic stress when compared to their single inoculations and untreated control. Equally, significantly reduced wilt incidence caused by *Ralstonia solanacearum* and higher fruit yield were observed when talc-based consortium formulation of *Trichoderma parareesei* + *Pseudomonas fluorescens* + *Bacillus subtilis* + *Azotobacter chroococcum* was applied onto tomato seeds (Nath et al., 2016). Besides, the co-inoculation can also have a negative impact on plant performance. According to Diniz et al. (2006), co-inoculation of *Trichoderma* spp., *B. bassiana*, *Metarhizium anisopliae*, and AM fungi greatly reduced the germination of lettuce seeds. Sometimes single inoculation can perform better than co-inoculation with several microbes. For instance, Ma et al. (2019) reported no benefit of *R. irregularis* applied *via* seed coating in combination with soil inoculated *Pseudomonas libanensis* on cowpea performance. On the contrary, when singly inoculated, *P. libanensis* was effective in enhancing cowpea biomass and seed yield. So far, it is not clear whether microbial consortia applied *via* seed coating can be advantageous. The most appropriate microbial combinations according to the plant species and growing conditions should be selected, and factors that affect the functioning of microbial consortia and their survival onto coated seeds must be investigated.

### Comparison of Seed Coating With Other Methods

Published data of comparisons between the efficiency and feasibility of inoculation of PBM *via* seed coating and other methods are still scarce. In a greenhouse experiment, after comparing seed coating of *Rhizobium* strains with soil drench application for the management of root-knot nematode *Meloidogyne incognita* on soybean, Ahmed et al. (2016a) found that seed dressing was more effective in controlling the reproduction of *M. incognita* and increasing plant height, and fresh and dry root and shoot weight. In a trial using maize, Rocha et al. (2019a) compared the delivery efficiency of *R. irregularis via* soil inoculation (4860 AM fungal propagules per plant) with seed coating (273 AM fungal propagules per seed) under greenhouse conditions. Results showed a similar root AM colonization between the two inoculation methods, despite the 20-fold difference in the amount of applied inocula. Schoina et al. (2011), in a greenhouse trial, evaluated the biocontrol efficacy of bacterial strain *Paenibacillus alvei* K-165 against the cotton phytopathogenic fungus *Thielaviopsis basicola* using the following: (1) seeds coated with a K-165 bacterial formulation in 10% xanthan gum-talc, (2) seeds coated with K-165 encapsulated in sodium alginate-Pyrax, and (3) solely K-165 encapsulated in sodium alginate-Pyrax pellets. Seed coating with K-165 xanthan gum and talc mixture was the most effective treatment in reducing disease symptoms and increasing plant height and fresh weight compared to sodium alginate-Pyrax encapsulated treatments. This might be due to the fact that coating with a bacterial formulation delivered higher bacterial concentration to the seeds and consequently to the rhizosphere, in comparison with other methods. In another study, Amutha (2017) compared four different inoculation methods (seed immersion, seed coating, foliar spray, and soil drenching) and found that all delivered *B. bassiana* to cotton plants, though with different levels of efficacy. Foliar application followed by soil drenching was considered the most effective inoculation method for *B. bassiana*. Müller and Berg (2008) tested *Serratia plymuthica* inoculation onto canola seeds using three different techniques (pelleting, film coating, and bio-priming) against *Verticillium* dahlia in greenhouse trials. Overall, *Serratia*-treated plants had significantly inferior disease severity compared to non-inoculated control, yet the efficiency varied with the employed technique. Film coating resulted in 5.2% disease suppression, while plants treated by pelleting and bio-priming showed 13.4% and 14.3%, respectively. In a field trial conducted by Rehman et al. (2018), *Pseudomonas* sp. MN12 was applied in combination with zinc (Zn) using four different methods (soil application, foliar spray, seed priming, and seed coating) to evaluate the interactive effect on wheat productivity. Results revealed that Zn application through any method including seed coating improved grain yield and grain Zn biofortification of bread wheat. Yet, maximum improvement of grain yield was recorded when Zn was applied in combination with strain MN12 through seed priming. The results from the above studies indicate that further investigations comparing different formulations and techniques can contribute to perfect seed coating. Notwithstanding, it is also important to ponder the economic feasibility of the method, since it can compromise large-scale applications.

### Agricultural Applications

In general, the application of microbial seed coating in agriculture is aimed at improving crop productivity. Seed coating with PBM has been successfully applied to a wide range of seeds with many different sizes, shapes, textures, and germination types (**Figure 1**). The most explored agricultural crops regarding inoculation *via* seed coating are cereals like wheat and maize, and fruit/vegetable crops such as tomato, cucumber, and sugar beet. Soybean, chickpea, and pea are some of most commonly reported oil and seed pulses crops. In addition, fiber crops like cotton or forage crops like alfalfa have also been addressed in PBM seed coating research.

In most reported studies, application of PBM *via* seed coating is able to promote crop growth (Sharma et al., 2003; Geetha and Balamurugan, 2011; Choi et al., 2016; Lally et al., 2017; Rozier et al., 2017; Accinelli et al., 2018b) or biocontrol of phytopathogens (Massoud et al., 2000; Anjaiah et al., 2006; Perelló et al., 2006; Haikal, 2008; Heo et al., 2008; Xue et al., 2013; Ahmed et al., 2016b).

#### Crop Production and Nutrition

Seed inoculation can improve not only plant growth and yield, but also nutritional value of the crops. Recently, Rouphael et al. (2017) evaluated two-seed propagated artichoke cultivars “Romolo” and “Istar” regarding planting time and seed coating with a consortium of AM fungi (*R. intraradices* and *F. mosseae*) and *T. atroviride*. They found that microbial seed coating improved both plant yield and nutritional value (such as antioxidant activity, total phenolics, caffeoylquinic acids, and flavonoids). The results showed that coating seeds with a consortium of PBM could assist host plants to achieve optimal yield with high nutraceutical properties when in combination with appropriate cultivar selection and agronomical practices. The increase in grain yield and yield stability with seed coating treatment was associated with higher nutrient uptake, soil plant analysis development index, and photochemical activity of photosystem II. The seed coating formulation with the above-mentioned AM fungi and *Trichoderma* consortium was based on previous results reporting enhancement of productivity of winter wheat and vegetable crops. In Colla et al. (2015a), the same consortium was inoculated *via* seed coating and significantly improved seedling growth (increase of 23%, 64%, and 29% in shoot and root biomass and the number of leaves, respectively), yield (increase of between 8.3% and 32.1%, depending on the growing season), and grain quality (increase of 6.3% in protein concentrations and general increase in K, P, Fe, and Zn concentrations) of winter wheat. When inoculated to the soil in the form of tablets, the same consortium of PBM increased the shoot dry weight by 167%, 56%, 115%, 68%, and 58% of lettuce, melon, pepper, tomato, and zucchini, respectively, in greenhouse experiments, and the shoot and root dry weight of lettuce by 61% and 57%, respectively, and the yield of zucchini by 15% under field conditions (Colla et al., 2015b). Seed coating with PBM can be particularly pertinent in low-input agriculture, due to its potential to reduce the application of fertilizers and improve food nutritional value. Oliveira et al. (2016a) showed that a silicon dioxide-based seed coating was a successful tool to inoculate the AM fungal isolate *R. irregularis* BEG140 that increased dry weight of shoot and seed spikes and nutritional contents (K and Zn) of wheat under reduced fertilization. The same coating formulation was used by Rocha et al. (2019a), where maize was grown without fertilization. Single inoculation with *R. irregularis* resulted in shoot nutrient concentration increments of 110%, 93%, 88%, and 175% for N, P, K, and Zn, respectively. In fact, the efficacy of some microbial inoculants for improving plant growth and yield can be influenced by nutrients addition/presence. In the study of Shaharoona et al. (2008), two ACC deaminase-producing *P. fluorescens* strains were coated with peat onto wheat seeds. Both pot and field trials revealed that the efficacy of *P. fluorescens* for improving growth and yield of wheat decreased with increasing rates of NPK added to the soil. Results showed that the right combination between proper doses of fertilizer and *P. fluorescens* could be used to improve plant growth while reducing fertilizer application.

#### Biocontrol

BCA and inducers of systemic acquired resistance (SAR) have been studied in order to reduce the use of fungicides in agricultural crops. Perelló and Bello (2011) evaluated the effectiveness of two *T. harzianum* strains (Th1 and Th2) and two synthetic compounds (acibenzolar-S-methyl and thiamethoxam) on wheat growth and suppression of tan spot caused by the fungal pathogen *Pyrenophora tritici-repentis*. Both biological and chemical agents were considered as SAR inducers. While acibenzolar-S-methyl solution was sprayed on wheat leaves, *Trichoderma* and thiamethoxam were coated onto seeds. Field trials showed that both biological and chemical agents can generally reduce the severity of tan spot, increasing plant height and weight in comparison with control. Th1 was responsible for reducing the presence of necrotic lesions (>50%), increasing foliar fresh weight (50%), and dry mass (25%). Activation of SAR in plants can be an alternative to maintain crops healthy and vigorous. The right combination of SAR inducers applied *via* seed coating with reduced rates of appropriate fungicides is a promising option for farmers. Further studies showed that the efficacy of plant disease control of fungicides and BCA applied *via* seed coating can be comparable. Mahmood et al. (2015) found that in a greenhouse study, both fungicides and BCA are almost equally effective against the chickpea wilt pathogen *F. oxysporum*. A treatment combining *T. harzianum* coated onto seeds with 1% methylcellulose solution and soil drench of fungicide carbendazim was proven to be more effective than individual treatment of the fungicide or the biocontrol agent. Mcquilken et al. (1990) showed that coating cress and sugar beet seeds with *P. oligandrum* oospores can control a range of damping-off diseases, in some cases, with the same efficiency as fungicide application. Seed coating with BCA could be used to reduce the amount of fungicide necessary to efficiently suppress disease in a susceptible cultivar. In some cases, the synergetic effect of BCA combined with reduced levels of fungicides can suppress disease equally to a fungicide application at full strength (Howell, 1991). Coating BCA onto agricultural crops can also be a viable, economical, and environmentally friendly strategy for weed control (Elzein et al., 2010). Elzein et al. (2006) showed that coating sorghum seeds with *Fusarium oxysporum* and gum arabic was an effective way to control the root parasitic weed *Striga*. They observed reductions of healthy emerged *Striga* shoots of 81% and 77% in sterilized and non-sterilized soil, respectively.

#### Abiotic Stress Tolerance

A small portion of the published research concerning PBM inoculation *via* seed coating is focused on improving crops resistance to abiotic stress. Recently, Rocha et al. (2019b) reported that coating cowpea seeds with *P. putida* using silicon dioxide and starch significantly increased biomass and seed yield under water deficit. The use of microbial inoculants is also considered as a promising option to enhance the production of cereals under salinity stress. Shahzad et al. (2017) showed that seed coating with *Bacillus* spp. improved gas exchange (e.g., photosynthetic rate, transpiration rate, and stomatal conductance), ionic content (e.g., N, P, and K of grain and straw), biochemical parameters (e.g., chlorophyll, carotenoids, and crude protein contents), growth, and yield attributes of wheat in saline soils. A greenhouse experiment using chickpea seeds coated with *Paenibacillus lentimorbus* B-30488 in combination with sodium alginate and calcium chloride (CaCl_2_) increased germination percentage and the number of colony-forming units of B-30488 in the rhizosphere, resulting in amelioration of drought stress by positively influencing the dehydration-induced physiological responses (Khan et al., 2011). The study revealed the potential role of sodium alginate and CaCl_2_ in affecting the biofilm formation of B-30488, and its adequacy for seed coating formulation in stress adaptation and protection of plants under drought stress.

#### Bio-Priming

Bio-priming is a process of biological seed treatment that combines seed hydration and seed inoculation with PBM to accomplish seed protection against soil-borne pathogens improving germination, seedling establishment, and vegetative growth (Meena et al., 2017). It is commonly used for biocontrol purposes. The inoculation of PBM in bio-priming can be done either by soaking seeds into a microbial suspension or by seed coating. In a study by Srivastava et al., 2010, tomato seeds were bio-primed by seed coating with inoculum of *T. harzianum* and *P. fluorescens* (either singly or in combination) using a slurry of talc (carrier) and gum arabic (binder). Application of *T. harzianum* and *P. fluorescens* by seed bio-priming significantly decreased the time needed for germination, increased germination rate, and reduced the incidence of *Fusarium* wilt in pot and field trials. The combinations of inoculants were more effective than single-isolate treatments. Pill et al. (2009) tested non-primed and primed slurry-coated cucumber seeds with commercial preparations of *T. harzianum* on seedling emergence and growth in *Phythium aphanidermatum*-infested growth medium. While *T. harzianum*-coated primed seeds had higher seedling emergence and seedling shoot fresh weight, non-primed *T. harzianum*-coated seeds displayed low incidence of damping-off caused by *P. aphanidermatum*. Rao et al. (2009) showed that coating and priming *P. fluorescens* onto sunflower seeds increased the control effect against *Alternaria* blight.

#### Limitations and Inconsistencies

Benefits of microbial seed coating on crop yield can be of short-term or null according to the growing conditions (Kubota et al., 2008). In fact, not all published research shows positive effects on plant performance of PBM inoculation *via* seed coating. No beneficial effect on crop productivity, nodulation, and biological N fixation (Knight, 2007), no economic gains when compared with fungicide application (Hartz and Caprile, 1995) and reduced biocontrol effect (Kay and Stewart, 1994) of inoculated seeds have been reported. For example, Diniz et al. (2009) coated sweet pepper seeds with a mixture of PBM (*Trichoderma viride*, *T. polysporhum*, *T. stromaticum*, *B. bassiana*, *M. anisopliae*, and AM fungi) and observed a negative impact on germination rate and plant height. The same undesirable effects were described regarding germination rate of lettuce seeds coated with the same mixture of PBM (Diniz et al., 2006).

**Figure 2:**
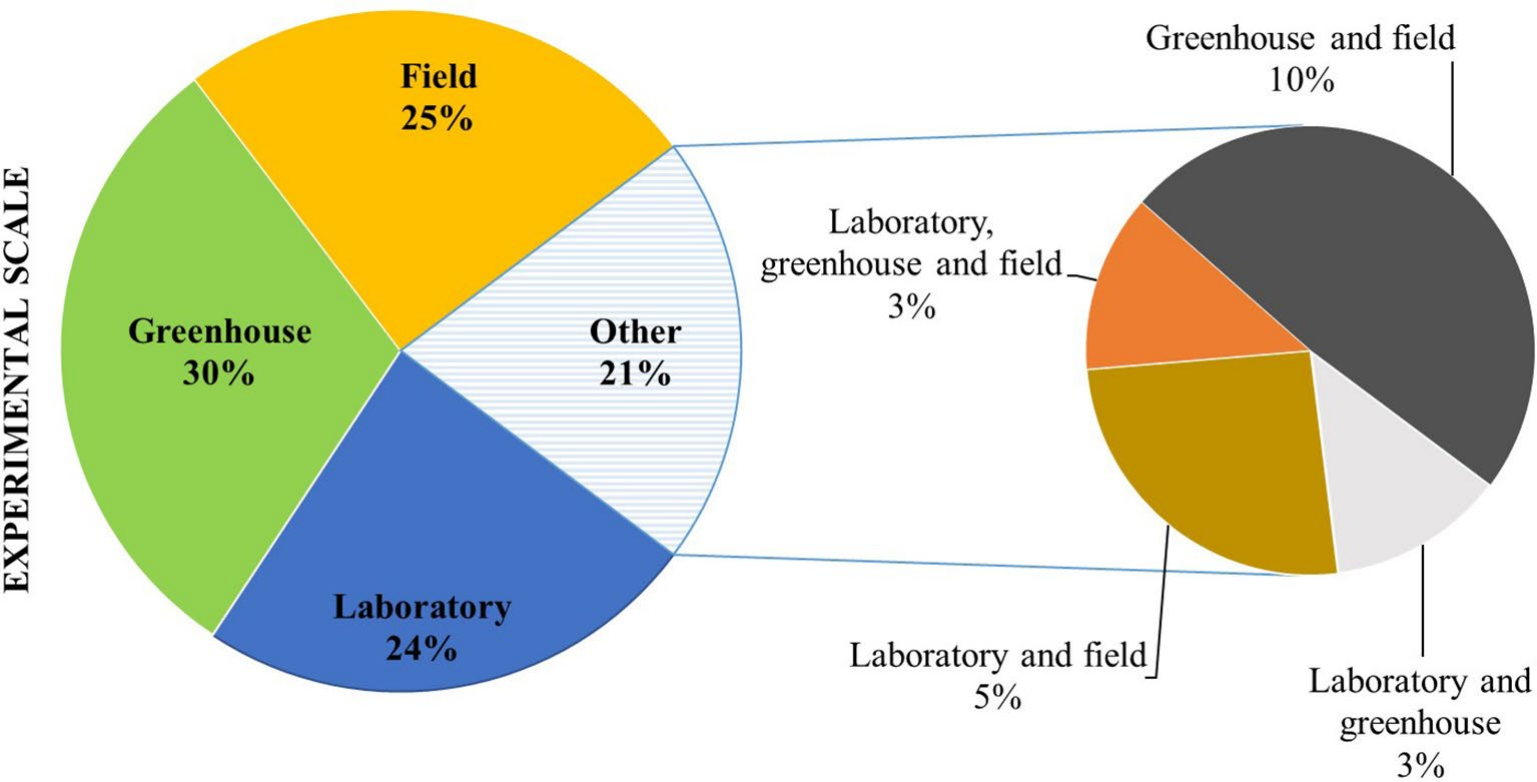
Scale of experiments of seed coating inoculation, expressed as percentage of studies (from a total of 191 papers published between 1960 and 2019).

Studies on microbial seed coating have been conducted in a similar proportion under laboratory, greenhouse, and field conditions (**Figure 2**). Still, only a small number of reports include all scales (e.g., laboratory, greenhouse, and field). Inconsistency of field performance can be one of the main restraints for the wide application of seeds coated with PBM. Thus, results that clearly validate the efficacy of the delivery system and the microbial application covering all stages of the process are essential. Shaharoona et al. (2006) tested the effect of ACC deaminase containing *Pseudomonas* spp. inoculated onto maize *via* seed dressing on plant growth in pot trials. The most efficient strains in promoting plant height, root weight, and biomass of maize were selected and tested under field conditions. Results indicated that rhizobacteria containing ACC deaminase are effective in improving growth and yield at low levels of fertilizer. Shaharoona et al. (2008) validated the positive effects of ACC deaminase producing *P. fluorescens* on growth, yield, and nutrient use efficiency of wheat under reduced levels of NPK in both pot and field trials. According to Anjaiah et al. (2006), *Pseudomonas* sp., *Bacillus* sp., and *Trichoderma* spp. inoculated onto groundnut *via* seed dressing were successfully used for biocontrol of pre-harvest seed infection by *Aspergillus flavus* under both greenhouse and field conditions.

The efficacy of microbial application methods may also vary according to the experimental scale. For instance, Kazempour (2004) evaluated the ability of *P. fluorescens* isolates to inhibit *R. solani* in rice under greenhouse and field conditions using different inoculation methods (seed coating, soil drenching, and foliar spray). *P. fluorescens* isolates were found to be more effective when delivered *via* seed coating under greenhouse conditions, while in the field, the best results were obtained with seed coating and foliar spray joint application.

**Figure 3:**
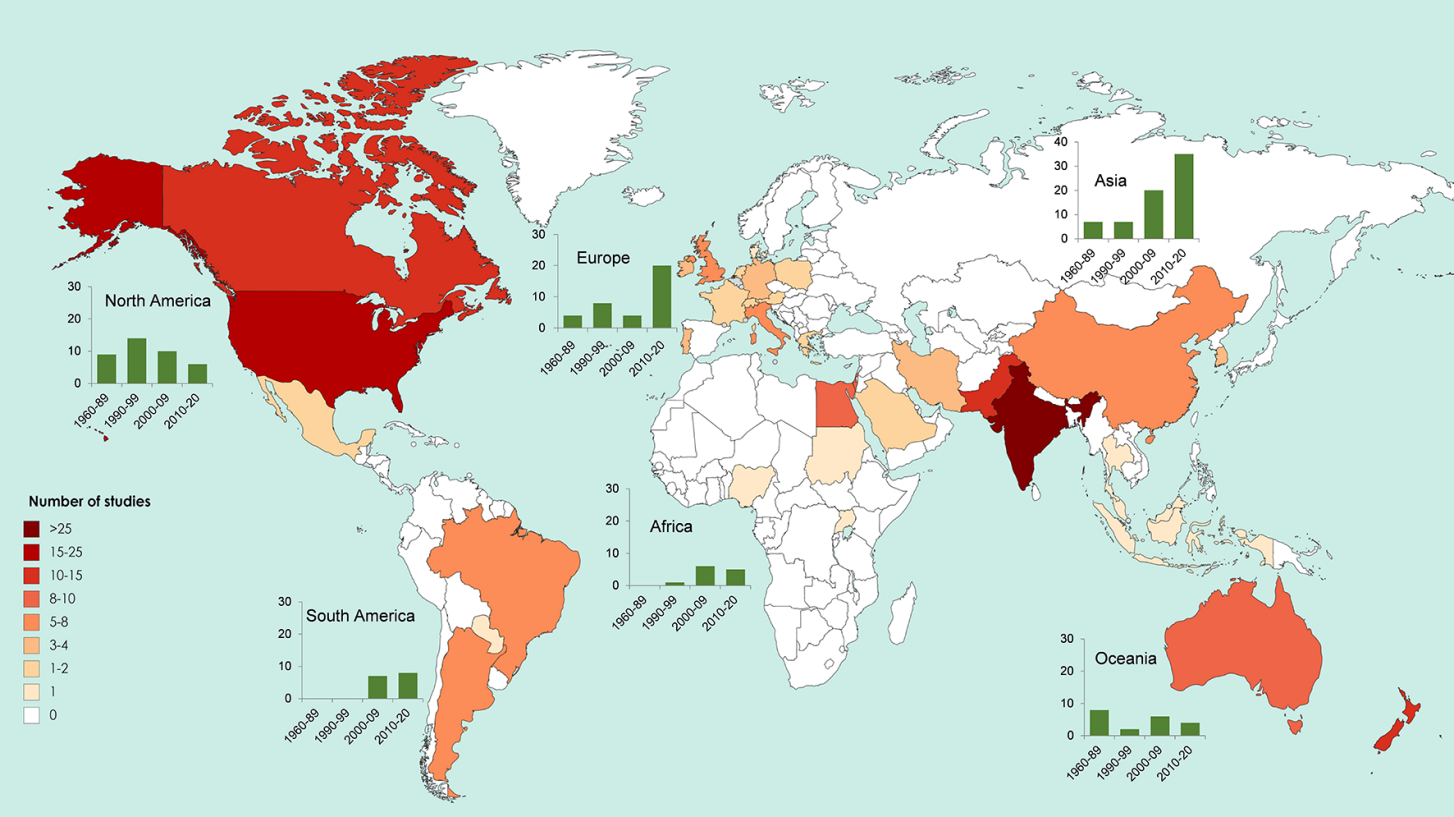
World map representing the number of studies dealing with seed coating with plant beneficial microbes by country and continent (from a total of 191 papers published between 1960 and 2019). Charts (green bars) indicate the number of published studies by continent, organized by intervals. Intervals correspond to decades (1990–99, 2000–10, and 2010–20), with the exception of the first interval, which collects studies of a larger period (1960–89) due to the low publication record during this period. Note the different scale of the y axis in the Asia chart.

Microbial seed coating is becoming more popular. From the 191 studies evaluated in this review, about 41% were developed over the last 9 years. This tendency is in accordance with the growing demand of the global market for biological seed treatment (Markets and Markets, 2018). **Figure 3** presents the distribution of studies regarding inoculation of PBM *via* seed coating worldwide. North American countries such as the United States of America and Canada, and from Asia like, India, and Pakistan, exhibited the higher number of studies. Nevertheless, Asian and European continents have the biggest increase in research regarding PBM seed coating during the last decade.

Noteworthy that microbial seed coating market will only reach its potential if bio-inoculants can be produced and applied in a cost-effective way and with efficient functionality regarding the purpose of application. Regardless of the abundant scientific literature on the capacity of several microbial inoculants to improve crop performance and tolerance to abiotic and biotic stresses, few of this work has been scaled up to commercial products or properly adapted for large-scale agricultural application.

## Conclusions and Future Opportunities

Driven by the need for sustainable and environmentally friendly farming practices and safer and healthier food, the demand for microbial inoculants is rising. Nevertheless, despite the well-known and proven benefits of PBM in improving yield, quality, and stress resistance of agricultural crops, few studies were focused on feasible delivery systems to apply microbial inocula in large-scale agriculture, frequently hampering its commercial exploitation and scale-up. Seed coating has the potential to be a cost-competitive and time-saving approach for crop production and protection, reducing application efforts and providing extra and desirable characteristics to the seeds. Yet, research on microbial seed coating has gaps that hinder its broader use. Research on inoculant formulation (e.g., microbial survival and viability, selection of ingredients, production cost) is still poorly explored. Even when considering the same type of ingredients (e.g., biochar), it is important to consider the chemical and physical properties that can differ and dictate the efficiency of the coating material as filler/carrier. On the other hand, although some materials (e.g., alginate) have the ability to increase microbial survival, they can also hamper germination, reduce shelf life of the coated seeds, or increase the cost of the final product. Choosing the right formulation for microbial inoculants can be truly challenging in seed coating; thus, more studies comparing different coating materials with different crops and microbes should be further explored. Overall, more clarity regarding the equipment and methodological details of seed coating with PBM is required. The combination of different PBM can also be challenging, since the functioning of consortia and their survival onto coated seeds are influenced by the plant species and growing conditions. The scarcity of studies comparing seed coating with other inoculation methods does not allow acknowledgement of the most suitable method to be used for microbial inoculation, yet some indicate that seed coating is very promising. Studies showed that selected PBM coated onto seeds can help to reduce the need for fertilizers and pesticides or to improve plant resilience to abiotic stress. Benefits of microbial seed coating for agricultural purposes are not always guaranteed, varying with the plant species, growing conditions, or experimental scale. Nonetheless, application of PBM *via* seed coating is gaining ground and claiming for more research and collaboration between seed companies and academics in order to overcome the technological and commercial hindrances.

The future of microbial seed coating is related to formulations that best adjust to local growing conditions and to agricultural practices (e.g., use of pesticides/fertilizers, irrigation management). Microbial formulations that compare and include native strains under local conditions and agricultural practices should be further explored as the awareness of potential risks by inoculation of non-native microbes is growing. Besides being able to improve microbial survival, coating materials (e.g., carriers and binders) could improve the performance/of the target crop. Since different PBM (species, strains, or isolates) could react differently to coating, the development of coating materials that are compatible with a wide range of inoculants could be crucial to the industry. Thus, new eco-friendly ingredients (e.g., compost; residues from forest and agriculture) with benefits to crops and soil should be considered for novel seed coating microbial formulations. In addition, studies on the economic viability of seed coating, including spending and gains (e.g., increased yield, reduction of fertilizers/pesticides and irrigation costs), should be conducted.

On the other hand, with the unavoidable climate change scenario, the roles of PBM in alleviating abiotic stress conditions (i.e., drought and salinity) of crops become of great importance and are promising lines of research for seed coating. In addition, the interest in areas such as ecosystem restoration is growing (as a result of the environmental degradation and climate change), which can also represent an interesting opportunity for seed coating as a microbial delivery tool. Therefore, revegetation of disturbed areas, conservation, or reintroduction of native plant species using microbial inoculation *via* seed coating are practices worth exploring.

The microbial inoculants have certainly huge potential for future agriculture good practice. However, it is important to assure that they are successfully applied in order to fulfill their role in a more sustainable agriculture. Seed coating is a potential tool for that chore. Further development and investment may allow its wider application and integration in agricultural management strategies, both in developed and developing countries.

## Author Contributions

RO and IR conceived the original idea. IR, PS-A, YM, and RO developed the manuscript structure and all the body text. RO and MV verified and supervised this work. All authors contributed to manuscript revision, read, and approved the submitted version. HF verified the final version of the manuscript.

## Funding

IR acknowledges the support of the Portuguese Foundation for Science and Technology (FCT) through the research grant SFRH/BD/100484/2014, the European Social Fund, and Programa Operacional do Capital Humano (POCH). This work was supported by the European Structural and Investment Funds in the FEDER component through the Operational Competitiveness and Internationalization Programme (COMPETE 2020) [Project No. 016801 (PTDC/AGR-TEC/1140/2014); Funding Reference: POCI-01-0145-FEDER-016801], and national funds through the Foundation for Science and Technology under the Project PTDC/AGR-TEC/1140/2014. This work was also financed by FCT within the project UID/BIA/04004/2019. MV acknowledges support by the Czech Academy of Sciences within the long-term research development project no. RVO 67985939.

## Conflict of Interest

The authors declare that the research was conducted in the absence of any commercial or financial relationships that could be construed as a potential conflict of interest.
